# Analysis of SARS-CoV-2 Variants From 24,181 Patients Exemplifies the Role of Globalization and Zoonosis in Pandemics

**DOI:** 10.3389/fmicb.2021.786233

**Published:** 2022-02-07

**Authors:** Philippe Colson, Pierre-Edouard Fournier, Hervé Chaudet, Jérémy Delerce, Audrey Giraud-Gatineau, Linda Houhamdi, Claudia Andrieu, Ludivine Brechard, Marielle Bedotto, Elsa Prudent, Céline Gazin, Mamadou Beye, Emilie Burel, Pierre Dudouet, Hervé Tissot-Dupont, Philippe Gautret, Jean-Christophe Lagier, Matthieu Million, Philippe Brouqui, Philippe Parola, Florence Fenollar, Michel Drancourt, Bernard La Scola, Anthony Levasseur, Didier Raoult

**Affiliations:** ^1^IHU Méditerranée Infection, Marseille, France; ^2^Microbes Evolution Phylogeny and Infections (MEPHI), Institut de Recherche pour le Développement, Aix-Marseille Université, Marseille, France; ^3^Assistance Publique-Hôpitaux de Marseille, Marseille, France; ^4^Vecteurs–Infections Tropicales et Méditerranéennes (VITROME), Institut de Recherche pour le Développement, Aix-Marseille Université, Marseille, France; ^5^French Armed Forces Center for Epidemiology and Public Health, Marseille, France

**Keywords:** SARS-CoV-2, variant, mutant, classification, travel, zoonosis, epidemics, pandemics

## Abstract

After the end of the first epidemic episode of SARS-CoV-2 infections, as cases began to rise again during the summer of 2020, we at IHU Méditerranée Infection in Marseille, France, intensified the genomic surveillance of SARS-CoV-2, and described the first viral variants. In this study, we compared the incidence curves of SARS-CoV-2-associated deaths in different countries and reported the classification of SARS-CoV-2 variants detected in our institute, as well as the kinetics and sources of the infections. We used mortality collected from a COVID-19 data repository for 221 countries. Viral variants were defined based on ≥5 hallmark mutations along the whole genome shared by ≥30 genomes. SARS-CoV-2 genotype was determined for 24,181 patients using next-generation genome and gene sequencing (in 47 and 11% of cases, respectively) or variant-specific qPCR (in 42% of cases). Sixteen variants were identified by analyzing viral genomes from 9,788 SARS-CoV-2-diagnosed patients. Our data show that since the first SARS-CoV-2 epidemic episode in Marseille, importation through travel from abroad was documented for seven of the new variants. In addition, for the B.1.160 variant of Pangolin classification (a.k.a. Marseille-4), we suspect transmission from farm minks. In conclusion, we observed that the successive epidemic peaks of SARS-CoV-2 infections are not linked to rebounds of viral genotypes that are already present but to newly introduced variants. We thus suggest that border control is the best mean of combating this type of introduction, and that intensive control of mink farms is also necessary to prevent the emergence of new variants generated in this animal reservoir.

## Introduction

Following its emergence in December 2019 in Wuhan, China, SARS-CoV-2 was declared to be a pandemic in March 2020 and its incidence since then has shown different epidemic patterns according to the country^1^^,^^2^ (Cucinotta and Vanelli, 2020). In Asia, a bell-shaped curve typical of seasonal viral respiratory infections has been observed. In Western developed countries, successive phases have occurred that have associated an initial bell-shaped curve and additional waves with one or more peaks. In Marseille, France, after a first epidemic period characterized by a bell-shaped curve of incidence followed by a period of almost zero incidence in May and June 2020, an epidemic burst of SARS-CoV-2 cases occurred in July. As this differed from the single bell-shaped curve typically observed for most respiratory viral infections, we suspected this corresponded to the introduction of new SARS-CoV-2 genotypes. Consequently, in the summer of 2020 we expanded our assessment of viral genetic diversity using genome next-generation sequencing. This allowed us to detect and report as early as 7 September 2020 several SARS-CoV-2 lineages that occurred concurrently or successively, and were associated with different genetic, epidemiological and clinical features (Colson et al., 2020b,2021c; Fournier et al., 2021).

These observations required defining and naming these different lineages to communicate about them, which is the very principle of taxonomy. Classifying and naming emerging infectious agents is essential (Sokal, 1974) but the task is particularly difficult in the case of SARS-CoV-2 for several reasons. First, as for other RNA viruses, SARS-CoV-2 evolves through continuous genetic variation with a high mutation rate (Eigen, 1996; Domingo and Perales, 2019). Second, SARS-CoV-2 has caused a dramatically high number of infections (over 195 million human cases worldwide and 5.9 million cases in France by 31 July 2021 (see footnote 2). It has been estimated that the SARS-CoV-2 genetic diversity in an infected patient may cover every single nucleotide substitutions and every possible pairs of nucleotide substitutions (Sender et al., 2021). In addition, there are animal reservoirs, a major one being mink (Fenollar et al., 2021; Oude Munnink et al., 2021). SARS-CoV-2 is, therefore, currently a rapidly evolving virus with a mutation rate estimated to be 9.8 × 10^–4^ substitutions/site/year (van Dorp et al., 2020) and characterized by a high rate of lineage turnover (Rambaut et al., 2020). Several SARS-CoV-2 specific classification and naming systems exist including those of GISAID^3^ (Alm et al., 2020), Nextstrain^4^ (Hadfield et al., 2018; Aksamentov et al., 2021), Pangolin^5^ (Rambaut et al., 2020), or the World Health Organization (WHO)^6^. But, although these are of great interest and usefulness, other classification and naming systems are also valuable, as previously acknowledged (Rambaut et al., 2020), particularly when it comes to better exploring genomic epidemiology on a local scale through real-time next-generation sequencing. Indeed, some classifications have become complicated with the advent of multiple new variants. In addition, local genetic trends may be underrepresented in the tremendous flow of available genomes. Moreover, correlations between genotypic patterns and epidemiological and clinical characteristics can be accurately performed at institutional or local levels through a large network of people who are responsible for patient diagnosis and clinical management, while remote collection and analysis of data may have poorer performance. Thus, we aimed at using our own definition, classification and naming of SARS-CoV-2 lineages and at correlating these lineages with our local epidemiological data in order to better investigate and monitor their genetic features and their sources and geographical origins. Here, we describe our classification and naming system of SARS-CoV-2 lineages and the history and characteristics of the major lineages detected among patients diagnosed at our institute, which sharply highlights the role of travel and globalization in the current pandemic.

## Results

### Broad Diversity According to Countries of the Patterns of Incidence of SARS-CoV-2-Associated Deaths

**FIGURE 1:**
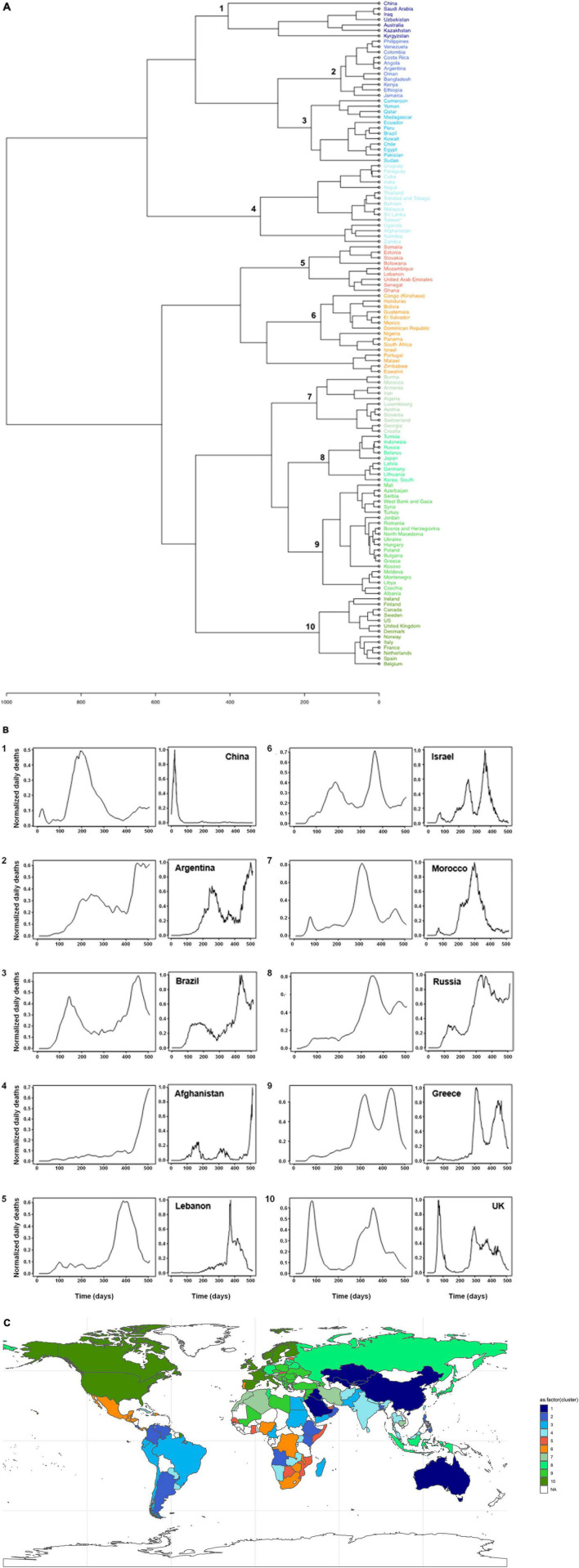
Patterns of incidence curves of SARS-CoV-2-associated deaths according to countries. **(A)** Hierarchical clustering of incidence curves of SARS-CoV-2-associated deaths per country. **(B)** Major patterns of incidence curves of SARS-CoV-2-associated deaths defined based on hierarchical clustering, and the example of one country for each pattern. Patterns of incidence curves of SARS-CoV-2-associated deaths are shown in 10 panels numbered from 1 to 10; these numbers correspond to those from the cladogram of panel **(A)**. The example of one country is shown in a panel at the right of each of the ten patterns. *X*-axis indicates time in numbers of days, and *Y*-axis indicates normalized daily deaths. **(C)** Worldwide distribution of the 10 major patterns of incidence curves of SARS-CoV-2-associated deaths defined based on hierarchical clustering. Colors are as used for panel **(A)**.

**FIGURE 2:**
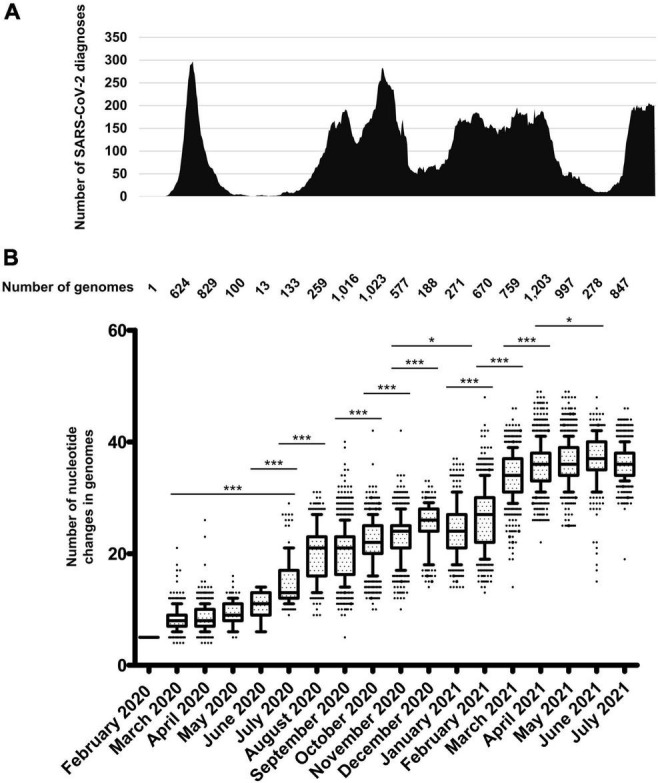
Chronological distribution of SARS-CoV-2 diagnoses by qPCR at IHU Méditerranée Infection institute **(A)**, and of mean (± standard deviation) numbers of mutations in SARS-CoV-2 genomes obtained from patients sampled per month **(B)**. **(A)** Chronological distribution of SARS-CoV-2 diagnoses is the mean number of diagnoses by qPCR per sliding windows of 7 days and steps of 1 day. **(B)** Numbers of mutations in SARS-CoV-2 genomes were calculated in reference to the genome of the Wuhan-Hu-1 isolate (GenBank Accession no. NC_045512.2). Whiskers indicate 10–90 percentiles. *p < 10^–1^; ***p < 10^–3^.

Clustering of the incidence curves of SARS-CoV-2-associated deaths for 221 countries delineated ten major groups of countries (Figure 1A). Each group comprised a mean ± standard deviation of 12 ± 4 countries (range, 7–21). A broad range of patterns was revealed through these groups, with countries experiencing one or more epidemic waves of varying intensity and duration combined or not with periods of lower and uneven incidence (Figure 1B). These data indicate that the incidence of SARS-CoV-2-associated deaths did not follow a single bell-shaped curve except in countries from Group 1, which includes China, Australia and a few countries in Central Asia and the Middle East. Instead, the incidence of SARS-CoV-2-associated deaths evolved elsewhere with multiple epidemic patterns according to time and geographic location. The worldwide distribution of these patterns showed that the majority of Western and Northern European countries (including France), the United States and Canada, which are part of Group 10, shared a same pattern (Figures 1A,C). Similarly, several countries in Asia and South America shared the same patterns leading to their classification in Groups 1–4. In contrast, a broad panel of patterns was observed for Africa. Countries that experienced a single peak of SARS-CoV-2-associated deaths (Groups 1, 4, 5, and 7) were mostly in Asia, Oceania, the Middle East, South America, Africa, and Eastern Europe; exceptions were Luxembourg, Austria and Switzerland. Among the countries which experienced several peaks of SARS-CoV-2-associated deaths, those in Group 10 were the developed industrialized countries of Western Europe and North America. These countries are particularly involved in globalization through tourism, business, and immigration (Chinazzi et al., 2020). In Marseille and its geographical area, the overall pattern of incidence of SARS-CoV-2-associated deaths was similar to that of countries in Group 10, which includes France, with a bell-shaped curve in March–April 2020 then a short period with a very low incidence followed by a longer period with two peaks of incidence and erratic incidence between these peaks (Figure 2A). This pattern of incidence with several peaks suggests multiple introductions of new variants that evolved abroad and caused distinct epidemics.

### Increase of SARS-CoV-2 Genetic Diversity Over Time

**FIGURE 3:**
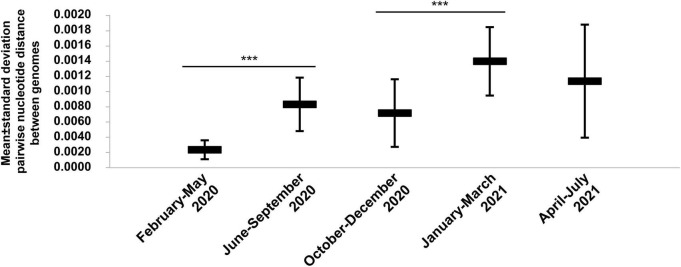
Chronological distribution of mean (± standard deviation) pairwise genetic distances according to time periods for SARS-CoV-2 genomes obtained from patients SARS-CoV-2-diagnosed at IHU Méditerranée Infection, Marseille. ***p < 10^–3^.

A total of 54,703 (10.6%) out of 513,805 patients tested in our institute for SARS-CoV-2 infection between 29 January 2020 and 18 August 2021 were positive by real-time reverse transcription-PCR (qPCR)^7^ (Figure 2A). We observed a bell-shaped curve of incidence between late February and early May 2020. This was followed by a period with almost no diagnoses (between 0 and 8 per day; mean ± standard deviation, 2.5 ± 2.2 per day) which lasted from 9 May to 5 July 2020. We performed whole SARS-CoV-2 genome sequencing using next-generation sequencing procedures since we diagnosed the first infection on 27 February 2020 (Colson et al., 2020a) and primarily obtained viral genomes from 309 patients for the March-April period (Colson et al., 2020b; Levasseur et al., 2020). In July 2020, the incidence of SARS-CoV-2 diagnoses increased again (Figure 2A). As the occurrence of a second epidemic 2 months after the end of the first one was unexpected for a respiratory virus infection, we suspected that it was linked to the introduction of new SARS-CoV-2 genotypes. This led us to quicky expand the next-generation sequencing of SARS-CoV-2 genomes to test this hypothesis. We primarily obtained 382 additional genomes during summer 2020 (Colson et al., 2020b), and ultimately analyzed the viral genomes obtained from a total of 9,788 patients sampled between 27 February 2020 and 18 July 2021. We observed a significant increase in the mean number of mutations in SARS-CoV-2 genomes between April 2020 and July 2020 [mean (±standard deviation) number of mutations per genome: 8.5 ± 2.0 versus 15.0 ± 4.4, respectively; *p* < 10^–3^ (ANOVA test)] (Figure 2B and Supplementary Table 1). This mean number continued to increase during the summer of 2020, and the increase was significant between July and August 2020 (20.0 ± 5.0). Thereafter, significant increases in the mean number of mutations per genome were observed between September and December 2020, (25.2 ± 4.3), between November 2020 (23.0 ± 4.1) and February 2021 (26.4 ± 5.7), and between February and March 2021 (34.1 ± 4.2). Similar trends were observed for the number of amino acid changes in the spike protein (Supplementary Figure 1). Overall, when analyzing the pairwise genetic distance between genomes obtained over time, two major periods with increased viral genetic diversity compared to previous months were identified, during the summer of 2020 compared to before June 2020, mean ± standard deviation of pairwise genetic distances between genomes being 8.28 × 10^–4^± 3.50 × 10^–4^ versus 2.34 × 10^–4^± 1.24 × 10^–4^, respectively (*p* < 10^–3^), then in January-March 2021 when the mean ± standard deviation pairwise genetic distances between genomes was 13.93 × 10^–4^± 4.48 × 10^–4^ compared to 7.14 × 10^–4^± 4.44 × 10^–4^ in October–December 2020 (*p* < 10^–3^) (Figure 3).

### The Marseille IHU Méditerranée Infection Classification System of SARS-CoV-2 Variants

Since the summer of 2020, we intensified our activity of SARS-CoV-2 genotyping using next-generation sequencing and also through performing partial spike gene sequencing and implementing variant-specific qPCR (Bedotto et al., 2021a,b). To characterize the increasing SARS-CoV-2 genome diversity observed during the summer of 2020, we promptly implemented a viral genome classification system and a nomenclature of SARS-CoV-2 variants. The term “SARS-CoV-2 variant” had scarcely been used in the scientific literature before September 2020 (Supplementary Figure 2) and no definition had been proposed to differentiate variants from mutants. We first reported the emergence of SARS-CoV-2 variants on 7 September, 2020 (Colson et al., 2020b), which included Marseille-2 (later named B.1.177 according to Pangolin classification) and Marseille-4 (B.1.160) variants that were then reported in October 2020 to have emerged and, for the case of Marseille-2, to have spread across Europe (Hodcroft et al., 2021).

There are no universally recognized or used strategies and criteria to classify viruses below the species level, and only biological classifications and nomenclatures have been proposed (Fauquet et al., 2008; International Committee on Taxonomy of Viruses (ICTV), 2020; Box 1). Classification and naming systems have been specifically proposed for SARS-CoV-2 including those of GISAID (see footnote 3) (Alm et al., 2020), Nextstrain (see footnote 4) (Hadfield et al., 2018; Aksamentov et al., 2021), Pangolin (see footnote 5) (Rambaut et al., 2020), and the WHO (see footnote 6) (Box 2). However, we chose to implement our own classification system and nomenclature and to use names rather than only numbers. This approach fitted the viral genomic epidemiology in our geographical area, and was more understandable and easier to use, which is the very purpose of classification and nomenclature. Our strategy consisted in detecting all mutations in all genomes obtained from patients diagnosed in our institute and in assigning a dynamic nomenclature to track SARS-CoV-2 genetic patterns.

**Box 1. [b] Definition of virus groups at and below species level.Viral species: Defined according to a polythetic classification as monophyletic groups of viruses whose properties can be distinguished from those of other species by multiple criteria including increasingly genetic criteria (https://talk.ictvonline.org/taxonomy/w/ictv-taxonomy) (Fauquet and Stanley, 2005; Adams et al., 2013).**

Below the level of species, there are currently no universally recognized or used strategies and criteria to classify viruses (Fauquet et al., 2008; International Committee on Taxonomy of Viruses (ICTV), 2020).

**Viral clade:** Monophyletic group comprised by a common ancestor and all its descent. These are branches with an independent root that obey a Darwinian evolutionary process.

**Viral isolate:** Viral population obtained by culture, generally linked to a particular virus sample (Fauquet and Stanley, 2005).

**Viral strain:** Virus of a given species with stable and heritable biological, serological, and/or molecular characteristics, and/or links to particular hosts, vectors, or symptoms (Fauquet and Stanley, 2005; Van Regenmortel, 2007).

**Viral quasi-species:** The mutant clouds (or swarms) with closely related genomes generated according to a Darwinian evolutionary process. It includes the accumulation of mutations during the replication of RNA viruses through continuous genetic variation, with possible genetic rearrangement by genetic recombination or genome segment reassortment, followed by competition among the mutants generated and selected in a given environment (Eigen, 1971, 1996; Domingo et al., 1976; Andino and Domingo, 2015).

**Viral mutant:** The result of nucleotide substitutions (non-synonymous or synonymous), deletions or insertions. These nucleotide changes occur randomly, gradually, and spontaneously, according to the Darwinian model (Eigen, 1993; Koonin, 2009; Domingo and Perales, 2019) and possibly at sites critical for viral protein conformation, antigenicity, susceptibility to immune responses, and functionality, including ability to interact with other proteins and enzymatic properties (Rambaut et al., 2004; Harvey et al., 2021).

**Viral variant:** An isolate that differs slightly by its transmission mode or the symptoms it causes from a reference virus (Fauquet and Stanley, 2005; Fauquet et al., 2008) or whose consensus genome sequence differs from that of a reference virus (Fauquet and Stanley, 2005; Van Regenmortel, 2007; Fauquet et al., 2008). Notwithstanding, no definition of the term “variant” is universal or broadly accepted (Fauquet et al., 2008; Kuhn et al., 2013). See Marseille classification of SARS-CoV-2 variants in Box 2.

**Box 2. [t] Major SARS-CoV-2 classification systems.World Health Organization (WHO) classification (https://www.who.int/en/activities/tracking-SARS-CoV-2-variants/): Defines SARS-CoV-2 ‘Variants of Interest’ (VOI) and ‘Variants of Concern’ (VOC). VOI: viral genome has mutations with established or suspected phenotypic implications and either has been identified to cause community transmission/multiple COVID-19 cases/clusters or has been detected in multiple countries. VOC: a VOI demonstrated through comparative assessment to be associated with (i) increase transmissibility or detrimental change in COVID-19 epidemiology; (ii) increase in virulence or change in clinical disease presentation; or (iii) decrease in effectiveness of public health and social measures or available diagnostics, vaccines, and/or therapeutics. Nomenclature uses letters of the Greek alphabet (currently from Alpha to Theta as of 1 August 2021).**

**GISAID classification (https://www.gisaid.org/references/statements-clarifications/clade-and-lineage-nomenclature-aids-in-genomic-epidemiology-of-active-hcov-19-viruses/) (Alm et al., 2020):** Defines high-level phylogenetic clades informed by the statistical distribution of genome distances in phylogenetic clusters followed by merging of smaller lineages into major clades (eight currently) based on shared marker mutations. Nomenclature uses actual letters of marker mutations (S, L, V, G, GH, GR, GV, GRY).

**Nextstrain classification (https://nextstrain.org/blog/2021-01-06-updated-SARS-CoV-2-clade-naming) (Hadfield et al., 2018; Aksamentov et al., 2021):** Defines major clades that reach ≥ 20% global frequency for ≥ two months, ≥ 30% regional frequency for ≥ two months, or correspond to a ‘variant of concern’ (VOC). Nomenclature uses a year-letter naming system.

**Pangolin (for Phylogenetic Assignment of Named Global Outbreak LINeages) classification (https://cov-lineages.org/pangolin.html) (Rambaut et al., 2020):** Based on a maximum likelihood phylogenetic reconstruction with the following criteria: (i) Phylogenetic evidence of emergence from an ancestral lineage into another geographically distinct population; (ii) ≥ one shared nucleotide difference(s) from the ancestral lineage; (iii) ≥ five genomes; (iv) ≥ one shared nucleotide change(s) among genomes from the lineage; and (v) a bootstrap value > 70% for the lineage node. Regarding the nomenclature: a letter (A for the Wuhan-Hu-1 isolate) is given first, followed by numbers for a maximum of three sublevels after which new descendant lineages are given a new letter.

**Marseille classification of variants:** Defines groups of viral genomes carrying specific sets of ≥five mutations along the whole genome differentiating them from any other viral genomes and obtained from ≥30 different patients. Nomenclature uses our city name, “Marseille,” followed by a number according to the chronology of their description (e.g., Marseille-1, Marseille-2,…) or to one of their hallmark amino acid substitutions (e.g., Marseille-501).

**FIGURE 4:**
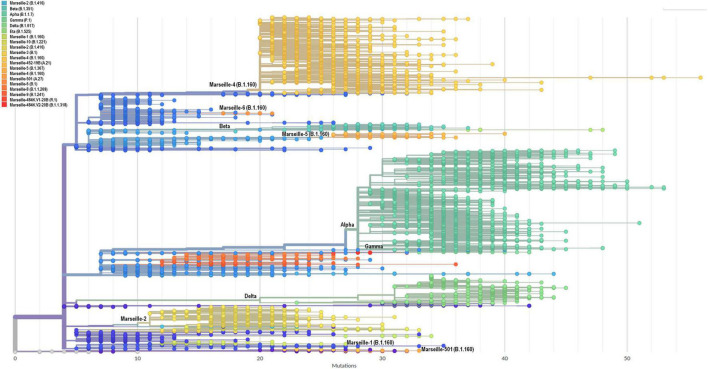
Phylogenetic tree of 10,773 genomic sequences of SARS-CoV-2 obtained from patients SARS-CoV-2-diagnosed at IHU Méditerranée Infection, Marseille, France. Phylogeny reconstruction was performed using the nextstrain/ncov tool (https://github.com/nextstrain/ncov) then visualized with Auspice (https://docs.nextstrain.org/projects/auspice/en/stable/). The genome of the original Wuhan-Hu-1 coronavirus isolate (GenBank accession no. NC_045512.2) was added as outgroup. Major (most prevalent) variants are labeled. Marseille variants or WHO clades, and Pangolin, or Nextstrain clades are indicated. *X*-axis shows the number of mutations compared to the genome of the Wuhan-Hu-1 isolate (GenBank accession no. NC_045512.2).

Phylogenetic trees were built that included all genomic sequences of SARS-CoV-2 obtained in our institute (Figure 4 and Supplementary Figure 3). Based on tree topology and to distinguish them from SARS-CoV-2 mutants, we delineated SARS-CoV-2 variants as groups of viral genomes carrying a set of at least five mutations along the whole genome differentiating them from any other viral genomes and obtained from at least 30 (initially 10) different patients. Genomes were, therefore, unambiguously assigned to a given variant based on the presence of particular sets of mutations (Table 1). This differentiated variants from mutants, the genomes of which do not harbor this minimal set of hallmark mutations. We first delineated seven Marseille variants of SARS-CoV-2 as early as 7 September 2020, 2 of which did not eventually reach the threshold number of 30 genomes (Colson et al., 2020b). This was the first description of an emergence and expansion of several SARS-CoV-2 variants responsible for distinct epidemics in a same geographical area. Three additional variants were then defined in 2020 (Supplementary Figure 4) and four in 2021, making a total of 12 (Table 1, Figure 4, and Supplementary Figure 4). In addition, we delineated 9 other viral lineages comprising genomes harboring a specific set of at least five mutations but obtained from < 30 different patients; some of these lineages may grow and become *bona fide* variants as additional members will be obtained. Besides, we detected four other SARS-CoV-2 variants that were defined as variants of concern (VOC; Box 2) and were named Alpha, Beta, Gamma and Delta by the WHO (see footnote 6). Regarding the nomenclature of the variants we defined, we used our city name, Marseille, followed by a number according to the chronology of their description (e.g., Marseille-1, Marseille-2,…) or one of their hallmark amino acid substitutions (e.g., Marseille-501). Hereafter, we will use both the Marseille and Pangolin nomenclatures to describe the genotypes of the SARS-CoV-2 genomes obtained in our institute.

**TABLE 1:** Nomenclature and description of Marseille (IHU Méditerranée Infection) variants of SARS-CoV-2.

Marseille variant (IHU Méditerranée Infection)	Number of genomes in the IHU Méditerranée Infection dataset	Nextstrain clade	Pangolin lineage	WHO classification	Mutations compared to the genome of the Wuhan-Hu-1 isolate (GenBank accession no. NC_045512.2)
**Defined**					
Marseille-1	88	20A	B.1.416	–	C28833T, G1181T, C1625T, C25886T, G28198T, G28851T
Marseille-2	617	20E (EU1)	B.1.177	–	T445C, C6286T, C22227T, C26801G, C28932T, G29645T, G21255C
Marseille-3	43	20A	B.1	–	C1912T, G5210A, C17470T, C21191T, A23148G, T27125C, C28854T
Marseille-4	2,442	20A.EU2	B.1.160	–	C18877T, C26735T, G5629T, G9526T, C11497T, G13993T, G15766T, G22992A, C25710T, T26876C, G28975C, G29399A, C4543T, A16889G, G17019T
Marseille-5	135	20C	B.1.367	–	C10582T, C28830A, C3099T, G4960T, C4965T, C6070T, C7303T, C7564T, C9246T, C10279T, C10301A, C10525T, G10688T, G11851T, C14230A, G21800T, G27632T, C27804T, G29402T, G29779T
Marseille-8	188	20B	B.1.1.269	–	C5055T, G11851T, A12755G, G24812T, C26895T
Marseille-9	114	20B	B.1.1.241	–	A11782G, T21570G, C21575T, T25473C, C28253T
Marseille-10	86	20A	B.1.221	–	C21855T, A25505G, G25906C, G25996T, C28651T, C28869T, C3602T, C6941T
Marseille-12	55	20A	–	–	G29734C, C17104T, T7767C, C8047T, C22879A
Marseille-501	47	19B	A.27	–	G28878A, G29742A, A361G, C1122T, C2509T, A9204G, A11217G, C16466T, A18366G, A20262G, T22917G, A23063T, C23520T, C23525T, G23948T, G25218T, T25541C, TTAATCCAG26159T, C27247T, TA27385T, AGATTTC28247A, A28273T
Marseille-484K.V3	84	21D	B.1.525	Eta	C1498T, A1807G, G2659A, C6285T, T8593C, GTCTGGTTTT11287G, C14407T, C18171T, A20724G, A21717G, C21762T, ATACATGT21764AT, TTTATTA21990TTTA, G23012A, G23593C, T24224C, C24748T, C26305T, T26767C, GTTT27204G, TCTG28277T, C28308G, A28699G, C28887T, G29543T, TTTCACCGAGGCCACGCGGAGTACGATCGAGTGTACAGTGAACAAT GCTAGG29727TGG
Marseille-452R-19B	51	19B	A.21	–	G28878A, G29742A, T1885A, C5907T, C10138T, G11417T, C11824T, C18129T, G18181A, T21982C, G22132T, T22917G, G23402A, G25687T, G26062T, C28253T, C29686T
**Potential**					
Marseille-6	11	20A	B.1	–	C9430T, A15477T, C18395T, A20622T, G20623T, A20624T, C23730T, A26319G, C28854T, G29044A
Marseille-7	17	20A	B.1.416.1	–	C28833T, C2706T, C25731T, G27463C
Marseille-11	19	20A	B.1.221	–	C21855T, A25505G, G25906C, G25996T, C28651T, C28869T, T445C, G13514T, G25785T, C25810T, G29511T, C29708T
Marseille-484K.V1-20B	1	20B	R.1	–	C14340T, G17551A, C18877T, A19167G, C19274A, T19839C, G22017T, G23012A, G23868T, T26604C, C27213T, TAAAA28270TAAA, C28833T, G29527T
Marseille-484K.V2-20B	9	20B	B.1.1.318	–	C3961T, C9072T, C9891T, C10116T, GTCTGGTTTT11287G, G20578A, C21846T, TTTATTA21990TTTA, G23012A, T23287C, C23604A, G23948C, C24382T, C25276A, T26767C, AAACGAACATGAAATTT27886AT, G28209T, A28271G, GCTA28895G, C29769T
Marseille-681H-19B	6	19B	A.23	–	G28878A, G29742A, G11230T, G22661T, G23401T, G28167A, G569A, C5178T, C7389T, C10029T, A15498T, C21646T, T23030C, C23604A, G25323T, C25603G, G25785T, T26767C
Marseille-681R-19B	7	19B	A.23.1	–	C9430T, A15477T, C18395T, A20622T, G20623T, A20624T, C23730T, A26319G, C28854T, G29044A
Marseille-4B	7	–	–	–	C18877T, C26735T, G5629T, G9526T, C11497T, G13993T, G15766T, G22992A, C25710T, T26876C, G28975C, G29399A, C1059T, C17634T, T19584C, G19684T, T25081C, G28378A, G29553T
Marseille-20E.2	14	–	–	–	C3646T, C6636T, C2862T, G5629T, A10652G, C13275T, C20844T, C21575T, G22104T, C24866T

### Emergence and Outcome of the SARS-CoV-2 Variants That Circulated in Our Geographical Area

#### Overall Prevalence of the Marseille SARS-CoV-2 Variants

SARS-CoV-2 genotyping was performed and successful for 24,181 (44%) of the 54,703 SARS-CoV-2-positive patients diagnosed in our institute as of 18 August 2021 (Table 2). SARS-CoV-2 genotyping was performed by genome sequencing in our institute for at least 12% of positive diagnoses of SARS-CoV-2 infection monthly, and for at least one fifth and one third of positive diagnoses monthly for 14 and 7 of the 18 months from February 2020 to July 2021, respectively (Supplementary Table 2). Genotype was obtained primarily by whole genome next-generation sequencing in 11,387 (47%) cases and by partial genome next-generation sequencing (spike fragment) in 2,621 (11%) cases, and retrospectively or prospectively (since January 2021) by in house variant-specific qPCR assays gradually designed and implemented subsequent to the detection of new variants (Bedotto et al., 2021a,b), in 10,173 (42%) cases. The most frequently detected variants were, in decreasing order, the Alpha (a.k.a. UK) variant, first detected in late December 2020 [Pangolin lineage B.1.1.7; *n* = 9,294 patients (38.4%)]; the Marseille-4 variant, first detected in July 2020 [B.1.160; 6,125 (25.3%)]; the Delta (a.k.a. Indian) variant, first detected in April 2021 [B.1.617.2; 4,113 (17.0%)]; the Marseille-2 variant, first detected in August 2020 [B.1.177; 890 (3.7%)]; and the Beta (a.k.a. South African) variant, first detected in January 2020 [B.1.351; 575 (2.4%)]. Other variants of significant prevalence and/or interest in our geographical area were the Marseille-8 variant [B.1.1.269; 176 (0.7%)]; the Marseille-5 variant [B.1.367; 124 (0.5%)]; the Marseille-1 variant [B.1.416; 123 (0.5%)]; the Marseille-9 variant [B.1.1.241; 117 (0.5%)]; the Marseille-484K.V3 variant [B.1.525; 112 (0.5%)], the Marseille-10 variant [B.1.221; 91 (0.4%)]; the Gamma (a.k.a. Brazilian) variant [P.1; 87 (0.4%)]; and the Marseille-501 variant [A.27; 74 (0.3%)].

**TABLE 2:** Numbers of the main SARS-CoV-2 variants and mutants diagnosed among the IHU Méditerranée Infection cohort of SARS-CoV-2-infected patients as of 18 August 2021.

Variant/ mutant name	Genotyping approach			Total	% of all genotypes
	Next-generation genome sequencing	Next-generation spike gene fragment sequencing	qPCR		
Alpha (B.1.1.7, a.k.a. UK)	3,506	2,220	3,568	9,294	38.44
Marseille-4 (B.1.160)	2,564	45	3,516	6,125	25.33
Delta (B.1.617.2, a.k.a. Indian)	1,406	0	2,707	4,113	17.01
Marseille-2 (B.1.177)	679	6	205	890	3.68
Beta (B.1.351, a.k.a. South African)	204	210	161	575	2.38
20A/8371T (1st period)	488	0	0	488	2.02
20A (1st period)	323	44	0	367	1.52
20C (1st period)	310	0	0	310	1.28
20B (1st period)	290	6	0	296	1.22
20A/15324T (1st period)	176	4	0	180	0.74
Marseille-8 (B.1.1.269)	174	2	0	176	0.73
Marseille-5 (B.1.367)	124	0	0	124	0.51
Marseille-1 (B.1.416)	123	0	0	123	0.51
20A/25563T (1st period)	123	0	0	123	0.51
Marseille-9 (B.1.1.241)	117	0	0	117	0.48
Marseille-484K.V3 (B.1.525)	112	0	0	112	0.46
20A/20268G (1st period)	95	0	0	95	0.39
Marseille-10 (B.1.221)	89	2	0	91	0.38
20A/2416T (1st period)	89	0	0	89	0.37
Gamma (P.1, a.k.a. Brazilian)	70	1	16	87	0.36
Marseille-501 (A.27)	26	48	0	74	0.31
Marseille-452R-19B (A.21)	43	2	0	45	0.19
Marseille-3 (B.1)	39	0	0	39	0.16
19B (1st period)	13	23	0	36	0.15
Marseille-12	36	0	0	36	0.15
20D (B.1.1.1)	31	2	0	33	0.14
20E (B.1.177)	21	0	0	21	0.09
Marseille-484K.V2 (B.1.1.318)	20	1	0	21	0.09
Marseille-11 (B.1.221)	19	0	0	19	0.08
19A (1st period)	15	0	0	15	0.06
B.1.214 (a.k.a. Belgian)	15	0	0	15	0.06
Marseille-7 (B.1416.1)	15	0	0	15	0.06
Marseille-6 (B.1)	9	0	0	9	0.04
Marseille-681R-19B (A.23.1)	7	0	0	7	0.03
Marseille-681H-19B (A.23)	6	0	0	6	0.02
Epsilon (B.1.427/B.1.429)	6	0	0	6	0.02
B.1.621	6	0	0	6	0.02
Marseille-484.V1 (A.23)	3	0	0	3	0.01

**Total**	**11,392**	**2,616**	**10,173**	**24,181**	**100.0**

#### Dynamics of SARS-CoV-2 Variants in the IHU Méditerranée Infection Cohort of SARS-CoV-2-Infected Patients

**FIGURE 5:**
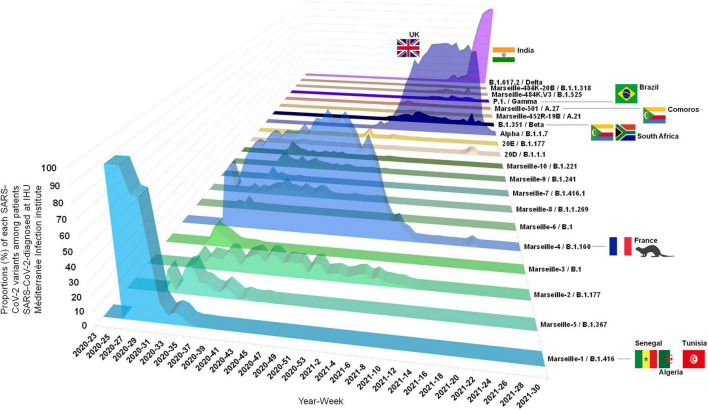
Three-dimensional plot of weekly proportions accounted by each SARS-CoV-2 variants among patients SARS-CoV-2-diagnosed at IHU Méditerranée Infection institute. See also IHU Méditerranée Infection (2021a; https://doi.org/10.35081/ffd4-1y77).

**FIGURE 6:**
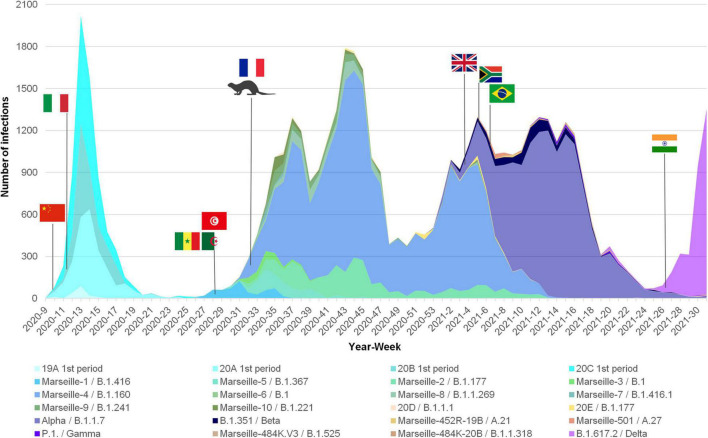
Weekly incidence of each SARS-CoV-2 mutants and variants extrapolated to the total number of cases, based on their proportions of genotyped cases, among patients SARS-CoV-2-diagnosed at IHU Méditerranée Infection institute.

**FIGURE 7:**
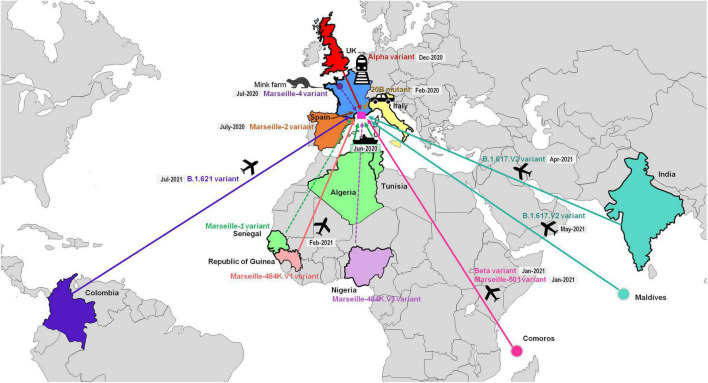
Demonstrated or likely sources and origins of SARS-CoV-2 mutants or variants detected among patients SARS-CoV-2-diagnosed in our institute. See also IHU Méditerranée Infection (2021b; https://doi.org/10.35081/nz2g-f980).

Based on our definition of variants, we were able to identify several since the summer of 2020, which we would currently call VOC (Box 2). After having defined and named the Marseille variants, we attempted to characterize their dynamic and to analyze their epidemiological features. For some of them, we were able to find their source. During the onset of the SARS-CoV-2 epidemic in late February 2020, we observed two distinct SARS-CoV-2 genotypes that did not make it possible to define variants based on our classification system. They corresponded to Nextstrain clades 19A and 20B that briefly co-circulated in our geographical area during the first period of the SARS-CoV-2 epidemic (Figures 5–7, Supplementary Figure 4, and Supplementary Table 3; IHU Méditerranée Infection, 2021a,b). The first clade was diagnosed from 15 patients in March 2020. The second, which corresponds to viruses harboring the D614G substitution in the spike, was initially retrieved from patients who stayed in northwest Italy and at the French/Italian border, and quickly established in the Marseille area as a majority clade together with clades 20A and 20C until May 2020.

We named the first variant we defined Marseille-1 (corresponds to Pangolin clade B.1.416), which emerged on week 27 of 2020 and predominated in July 2020 before rapidly disappearing at the end of August 2020 (Colson et al., 2021c; Figures 5–7, Supplementary Figure 4, and Supplementary Table 3; IHU Méditerranée Infection, 2021a,b). Its onset coincided with the resumption on 1 July 2020 of maritime connections between Marseille and the Maghreb. As 16 of the first 20 diagnosed cases (80%) were detected in people who had traveled by boat or on passenger boats circulating between the Maghreb and Marseille, we assumed that this variant was brought to Marseille from the Maghreb. Furthermore, genomic analyses showed that it originated from sub-Saharan Africa, mostly Senegal (Colson et al., 2021c).

The Marseille-4 variant (B.1.160) was the most prevalent SARS-CoV-2 variant in our geographical area in 2020 and was only outdone by the Alpha (UK, B.1.1.7) variant in 2021 (Fournier et al., 2021). It replaced the Marseille-1 variant in August 2020 and faded away in April 2021. It was first detected in south-west France and in Marseille. Its emergence coincided with the re-increase in France of SARS-CoV-2 incidence starting with a department in northwest France (Mayenne) close to the Eure-et-Loir department where a mink farm was found to be massively infected with SARS-CoV-2 (Fournier et al., 2021; Santé Publique France, 2021). The only genome from this farm, obtained from a mink sampled in mid-November 2020 but released in March 2021, is that of a Marseille-4 variant (GISAID Accession no. EPI_ISL_1392906). In addition, the co-occurrence of 13 hallmark mutations in the Marseille-4 variant without any available genome showing a gradual appearance of these mutations suggested that genetic evolution had been overlooked. These data led us to conclude, based on genomic, geographical and temporal coincidences, that the source of the Marseille-4 epidemic was the mink. The Marseille-4 variant was progressively replaced from January 2021 by the epidemic linked to the Alpha (UK, B.1.1.7) variant. Meanwhile, it circulated in 76 countries and accounted for 31,299 genomes in the GISAID database as of 30 June 2021, mostly obtained from European countries [in 29,906 cases (96%)] (Supplementary Figure 5).

During July and August 2020, two additional variants, Marseille-2 and Marseille 5, emerged in our geographical area, which became the second and third most prevalent variants from week 11 of 2020 after the Marseille-4 variant. The Marseille-2 variant (B.1.177) most likely emerged in Spain, where it accounted for two-thirds of released genomes and it spread to our geographical area and through Europe during the summer of 2020 (Hodcroft et al., 2021). The Marseille-5 variant (B.1.367) disappeared in October 2020 and its source and origin are unknown. The Marseille-8 (B.1.1.269) and Marseille-9 (B.1.1.241) variants were first detected in August 2020. They accounted overall for approximately 1.2% of the cases of SARS-CoV-2 infections in our geographical area, but nonetheless circulated until March and January 2021, respectively. The source and origin of these variants are unknown.

The Marseille-501 variant (A.27) emerged in Marseille in January 2021 as the first variant of lineage 19B harboring amino acid substitution N501Y in the spike protein (Colson et al., 2021b). A majority of the genomes primarily available from the GISAID database were linked to Mayotte, which is part of the Comoros archipelago located in the Indian Ocean from where originated some of the patients we diagnosed to be infected with this variant. The Marseille-484K.V3-20A variant (B.1.525) emerged in April 2021 in Marseille and was the majority variant apart from Beta (South African) and Gamma (Brazilian) variants to harbor amino acid substitution E484K in the spike protein. This variant was first described in Nigeria on 11 December 2020, but we have not documented its importation from this country. The Alpha variant (B.1.1.7) emerged in December 2020 in France and in Marseille and faded away in June 2021 after having been responsible for the majority of SARS-CoV-2 infections from February 2021. The first cases were part of a family cluster during the Christmas holidays after some family members came from England by train. We also had smaller epidemics with the Beta (South African, B.1.351) and the Gamma (Brazilian) (P.1) variants from January 2021. Regarding the Gamma variant, we first detected it among people returning from the Comoros who had traveled through Ethiopia then Tunisia to Marseille. Between February and April 2021, we also detected three cases of a rare spike E484K-harboring SARS-CoV-2, which we named Marseille-484K.V1 (A.23) (Colson et al., 2021a). The three genomes were phylogenetically closest to a genome from the Republic of Guinea (a.k.a. Guinea-Conakry), where one of the patients had traveled.

Most recently, we have mostly detected infections with the Delta (B.617.2) variant, in 4,113 patients. The two first patients diagnosed with this variant in late April 2021 were an Indian sailor re-embarking in Marseille after the resumption of maritime travel (La Scola et al., 2021), and a physiotherapist who had traveled to the Republic of the Maldives, in Southeast Asia, and provided care upon his return to France in an accommodation facility for dependent elderly people, being involved in a cluster among residents and health care workers. Finally, in mid-July 2021, we detected a variant classified in the Pangolin lineage B.1.621 (a.k.a. Mu), that was first identified in Colombia in January 2021 (Laiton-Donato et al., 2021). Information was available for one of the six patients infected with this variant, a 10-year-old Colombian girl who developed symptoms and was diagnosed in our institute 4 days after she arrived in France.

Overall, we identified several SARS-CoV-2 variants that were responsible as early as the summer of 2020 for distinct epidemics causing several waves of SARS-CoV-2 incidence (Figures 5–7; IHU Méditerranée Infection, 2021a,b). The epidemics caused by these different variants each exhibited a bell-shaped curve, and they occurred successively or concomitantly, therefore contributing to the total case load. We documented that new SARS-CoV-2 variants were introduced in our geographical area by boat in one case [Marseille-1 (B.1.416)], by train in one case [Alpha (B.1.1.7)], and by plane in five cases [Beta (B.1.351), Marseille-501 (A.27), Marseille-484K.V1 (A.23), Delta (B.1.617.2), and B.1.621 variants]. In addition, the importation of Marseille-484K.V3 (B.1.525) from Nigeria through air travel is suspected. Importation from abroad through travel therefore accounted for a substantial source of variants and, subsequently, of cases. The other source identified for the emergence of a new variants is the mink epidemics in Northern France, with the case of Marseille-4 (B.1.160) (Fournier et al., 2021).

## Discussion

In this study, we wanted to clarify the classification and naming of SARS-CoV-2 genotypes we implemented in our institute in order to have a simple identification of SARS-CoV-2 variants and to characterize their mutations, their source, and spread. This is essential to get a better understanding of SARS-CoV-2 epidemiology. Since the SARS-CoV-2 emergence in France, we have performed in our institute extensive surveillance, identification and monitoring of SARS-CoV-2 variants. Currently, the viral genotype has been determined for 44% of the patients diagnosed in our institute and the viral genome was obtained in approximatively one fifth of patients diagnosed with SARS-CoV-2. As early as the summer of 2020, we increased our surveillance of SARS-CoV-2 genotypes which allowed us to decipher several features of the current SARS-CoV-2 pandemic on a local scale. Before the summer of 2020, very few studies had pointed out an increase of SARS-CoV-2 genetic and amino acid diversity. Noticeably, Tomaszewski et al. (2020) had reported new pathways of mutational changes in SARS-CoV-2 proteomes. We used several approaches for SARS-CoV-2 classification as it can be determined based on a limited number of genetic markers (in the case of the spike gene fragment sequenced here) (Colson et al., 2021b) and even a single one (in the case of variant-specific qPCR) (Bedotto et al., 2021a,b). Whole genome analysis provides the most precise classification as it considers multiple genetic markers along the genomes and allows characterizing genetic diversity beyond lineage identification. However, variant-specific qPCR allows rapid classification at low cost and high throughput including in case of low viral load.

First, it appears relatively clear that in countries which have been isolated, either geographically (islands that have closed their access) or politically (China and Korea), there has been only one major epidemic episode with a bell-shaped incidence curve. In Europe and the United States, where such a policy has not been implemented or has been made impossible by political circumstances, several epidemics have occurred successively or concurrently with different viral variants. Marseille, which is a very particular city due to its geographical location, has for centuries been at the forefront of imports of epidemics including of plague and cholera (Barbieri et al., 2021), and operated in an identical way during the SARS-CoV-2 pandemic. Thus, we observed that the first diagnosed SARS-CoV-2 infections were imported cases involving people who had stayed in northwest Italy and at the French/Italian border. This is congruent with findings from a study that inferred the introduction of SARS-CoV-2 from Hubei province into Italy and France before early March 2020 and also estimated that viruses that spread during the epidemic onset in Europe had a common ancestor in Italy (Nadeau et al., 2021). This preceded a first epidemic phase during which international borders were closed. Their reopening in early summer 2020 was followed by a short epidemic. This was linked to a SARS-CoV-2 variant that we named Marseille-1 (B.1.416), which was imported from North Africa by boat and originated from sub-Saharan Africa, and was probably not very transmissible, since this epidemic ended 2 months after its emergence and did not spread beyond the Marseille region. Another variant, which we named Marseille-2 (B.1.177), was introduced to our geographical area during the summer of 2020 from Spain, where it appears to have originated (Hodcroft et al., 2021). Later, in 2021, the Alpha (B.1.1.7, or UK) variant was first imported in Marseille by French people returning from the UK. In addition, in the Marseille population which includes lots of people of Comorian origin, we observed the onset of small epidemics with the Beta (B.1.351, or South African) variant among people traveling by plane from the Comoros, and with the Marseille-501 (A.27) variant that involved people of Comorian origin including from Mayotte (Colson et al., 2021b). Similarly, the reopening of sea cruises in the spring of 2020 was associated with an importation of the Delta (B.1.617.2, or Indian) variant by an Indian sailor who had traveled from India to Marseille (La Scola et al., 2021), and a second case identified of this variant was in a traveler returning from the Republic of the Maldives located in the Indian Ocean. Two other SARS-CoV-2 variants were imported in 2021 from the Republic of Guinea and from Colombia. We therefore observed that the importation from abroad through travel accounted for a substantial source of variants and subsequently of infections, highlighting the role of globalization in the spread of SARS-CoV-2 pandemic. Marseille is a city with many immigrant adults and children, and its geographical location and harbor make it very open to travelers. This explains the importation of infectious agents of various origins, which has been recognized in Marseille since antiquity (Barbieri et al., 2021). The considerable role of travel in the introduction and subsequent spread of new SARS-CoV-2 variants on the national or continental scales has also been reported in a recent phylogeographical study that analyzed genomic, epidemiological, and mobility data (including ours) collected from ten European countries between January and October 2020 (Lemey et al., 2021). This study found that the intensity of international travel predicted the spread of SARS-CoV-2 with the introduction of new viral variants, and it estimated that more than half of the lineages that were spreading in late summer 2020 had been newly introduced to countries since mid-June 2020. Such importations into countries of SARS-CoV-2 variants and their impact on the pattern of incidence of SARS-CoV-2 cases have also been reported in other studies (McLaughlin et al., 2021). This sharply confirms what we described as early as at the beginning of September 2020 (Colson et al., 2020b) with the emergence of seven new SARS-CoV-2 variants including one [Marseille-1 (B.1.416)] for which we had already found a source (Colson et al., 2021c). Thus, we were the first to consider and highlight that the re-increase of SARS-CoV-2 incidence observed during the summer of 2020 in Marseille and in France may have resulted from the importation of new variants rather than from a rebound of previous viral genotypes (Colson et al., 2020b,2021c). This aligns with the fact that countries for which cross-border travel was the most limited geographically and/or politically by closing the borders experienced a single epidemic peak, whereas countries with high levels of international travel experienced several epidemic peaks and longer periods with a significant incidence of infections. Hence, these findings indicate that while local control measures such as lockdowns and curfews may limit viral spread, they are inefficient to avoid the introduction of new viral genotypes. The role of local lockdowns in controlling the viral spread has been controversial. This measure has been found to be irrelevant by some researchers (Melnick and Ioannidis, 2020). In Japan, lockdown was not associated with a reduction of SARS-CoV-2 incidence, whereas travel restrictions were (Hideki and Skidmore, 2021).

Second, our findings indicate that the role of epizootics in dense animal herds has been overlooked in the generation of variants, their transmission to humans, and their contribution to the SARS-CoV-2 pandemic. Mink was revealed as a major source of SARS-CoV-2 diversity and a key element in the expansion of the human pandemic (Fenollar et al., 2021; Oude Munnink et al., 2021). Data have accumulated indicating that the current pandemic, which is primarily a zoonosis, continued being a zoonosis with the massive involvement of minks, while other animals, including domestic animals such as cats and dogs, have also been affected (Fenollar et al., 2021). It has been demonstrated that minks were a source of new variants that fuelled the pandemic in Denmark (Oude Munnink et al., 2021), and SARS-CoV-2 spike protein gene variants harboring amino acid substitutions N501T and G142D were also found to circulate in both humans and minks in the United States (Cai and Cai, 2021). In Marseille, we accumulated indications that the Marseille-4 variant originated from mink. This hypothesis took us a long time to confirm due to the very late release, in March 2021, of the viral sequence obtained from a mink farm in Eure-et-Loir in mid-November 2020. Interestingly, the detection of two epidemics, in Marseille and retrospectively in Mayenne (Northwest France), appeared to be unrelated as the absence of SARS-CoV-2 genotyping initially carried out in Mayenne and Eure-et-Loir prevented the demonstration that they were due to the same variant and that virus transmission could have occurred from farm minks located in a neighboring department. It is worthy to note that the Marseille-4 variant was responsible for a large epidemic in France and also to a lesser extent in other European countries and in United Kingdom and was probably considerably overlooked as the global concern about SARS-CoV-2 variants only emerged several months after we described it. A recent study of global and regional adaptative evolution of SARS-CoV-2 delineated a divergent viral clade with a pattern of signature mutations consistent with the Marseille-4 variant. This clade was reported to have first appeared in Europe in April 2020 then to have considerably expanded during summer 2020 (Rochman et al., 2021).

These findings show that SARS-CoV-2 genomic surveillance and the definition of variants are valuable for a better assessment of SARS-CoV-2 epidemiology. In addition, they allow correlating viral genotypes with the clinical severity and outcome of infections. Indeed, several studies have reported different clinical patterns of infections according to the SARS-CoV-2 variant (Dao et al., 2021; Domingo et al., 2021). We previously compared the clinical symptoms and outcomes of patients infected with different variants we identified. Compared with viruses of the Nextstrain clade 20A that circulated between February and May 2020 in Marseille, patients infected with the Marseille-1 (B.1.416) variant were less likely to report dyspnea and rhinitis and to be hospitalized (Dao et al., 2021). In addition, patients infected with the Marseille-4 variant (B.1.160) were more likely to exhibit fever and to be hospitalized than those infected with the Marseille-1 variant, and they were also more likely to exhibit fever than those infected with a virus of Nextstrain clade 20A that circulated between February and May 2020 in Marseille. Also, patients infected with N501Y-harboring SARS-CoV-2 variants [namely, Alpha (B.1.1.7), Beta (B.1.351), and Gamma (P.1) variants] were less likely to be hospitalized than those infected with the Marseille-4 variant and with viruses of Nextstrain clade 20A that circulated between February and May 2020 in Marseille (Dao et al., 2021). SARS-CoV-2 genomic surveillance can also provide insights for phenotypic, structural and functional investigations of mutations and variants, as has been already broadly performed (Dong et al., 2021; Fantini et al., 2021; Gupta et al., 2021; Jaafar et al., 2021; Rahman et al., 2021). Particularly, SARS-CoV-2 mutation patterns in the spike protein may affect binding of neutralizing antibodies or T-cell recognition.

Furthermore, a key issue from the present study is highlighting that the fluctuations in incidence of SARS-CoV-2 infections that we observed resulted from the succession or combination of distinct epidemics caused by different viral variants rather than from the rebound of persisting lineages, which we primarily described in September 2020 and was confirmed at larger scale (Lemey et al., 2021). Since September 2020, these genetic evolutionary and epidemiological patterns continued to be observed and the surveillance of SARS-CoV-2 variants, the role of travel and globalization in their spread, and the turnover of major VOC has come to the frontline, particularly with the Alpha, Beta, Gamma, and Delta variants (Del Rio et al., 2021; Harvey et al., 2021).

Overall, previous findings have revealed that epidemic waves mostly observed in developed Western countries are, in fact, distinct epidemics due to different SARS-CoV-2 variants. Therefore, as the major identified sources for these SARS-CoV-2 variants is cross border travel and animal reservoirs, efficient border controls and surveillance of mustelid farms are critical in order to avoid the introduction of new SARS-CoV-2 variants and the occurrence of new epidemics.

## Materials and Methods

### Epidemic Curves for SARS-CoV-2-Associated Deaths in Various Countries and Clustering of the Dynamic of Epidemics at Country Scale

We used the cumulated daily mortality from the COVID-19 data repository operated by Johns Hopkins University (Dong et al., 2020) available at https://github.com/CSSEGISandData/COVID-19 (accessed on 25 June 2021), which covers 221 countries and locations from 22 April 2020 to 24 June 2021. Data mining of time series is a complex scientific field where the analysis process must be tailored to the characteristics of the series and the clustering objectives (Liao, 2005; Fu, 2011). Our objective was to cluster the dynamic of epidemics at country scale as reflected by COVID-19 mortality, taking in account the time of the different series peaks but not country mortality incidences. A threshold of 500 deaths was used for countries to analyze their SARS-CoV-2-associated death curves. We transformed the cumulative death counts into smoothed (7 days centered moving average) daily death rates. The resulting series were then clustered using the Pearson distance after normalization as L2-normed lock-step distance measure (Mori et al., 2016) and the ascendant hierarchical classification algorithm with Ward aggregation. For determining the resulting clusters, we used a dynamic cluster detection method (Langfelder et al., 2008) with a minimum cluster size set at five countries. We then mapped the geographic distribution of cluster members. The data mining process was done using R v4.0.2^8^ with the packages vegan, forecast, dynamictreecut, and rnaturalearth.

### Study Period and Clinical Samples

SARS-CoV-2 genotyping was performed from nasopharyngeal samples tested between 27 February 2020 and 18 August 2021 (18 months) at the IHU Méditerranée Infection institute^9^. Specimens with a cycle threshold value (Ct) of qPCR lower than 20 were selected as a in priority for whole genome sequencing of SARS-CoV-2 genomes, and those with a Ct between 20 and 30 were included secondarily to ensure a more comprehensive coverage of the study period. Partial sequencing of the SARS-CoV-2 spike gene generating a 1,854 nucleotide-long sequence was performed in 2020 and, mainly, between January and April 2021, as previously described (Colson et al., 2021b). For respiratory specimens with Ct values > 30 or those with Ct values < 30 but from which genome sequences were not obtained, we identified those harboring specific Marseille genotypes using qPCR targeting variant-specific regions, as previously described for the Marseille-1 variant (Colson et al., 2021c) and the Marseille-4 variant (Bedotto et al., 2021b; Fournier et al., 2021). Additional in house and commercial variant-specific qPCR assays were used for other variants. The study was approved by the ethics committee of the University Hospital Institute Méditerranée Infection under No. 2020-016-03.

### SARS-CoV-2 Genome Sequencing

Viral RNA was extracted from 200 μL of nasopharyngeal swab fluid using the EZ1 Virus Mini Kit v2.0 on an EZ1 Advanced XL instrument (Qiagen, Courtaboeuf, France) or the KingFisher Flex system (Thermo Fisher Scientific, Waltham, MA, United States), following the manufacturer’s instructions. SARS-CoV-2 genome sequences were obtained by next-generation sequencing with various procedures since February 2020 until August 2021: (i) with the Illumina technology using the Nextera XT paired end strategy on MiSeq instruments (Illumina Inc., San Diego, CA, United States) since February 2020, as previously described (Colson et al., 2021c); (ii) with the Illumina COVIDSeq protocol on a NovaSeq 6000 instrument (Illumina Inc.) since April 2021; or (iii) with Oxford Nanopore technology (ONT) on MinION or GridION instruments (Oxford Nanopore Technologies Ltd., Oxford, United Kingdom), as previously described (Colson et al., 2021c). Next-generation sequencing with ONT was performed without or with (since March 2021) synthesized cDNA amplification using a multiplex PCR protocol with ARTIC nCoV-2019 V3 Panel primers purchased from Integrated DNA technologies (IDT, Coralville, IA, United States) according to the ARTIC procedure^10^. After its extraction, viral RNA was reverse-transcribed using SuperScript IV (ThermoFisher Scientific) prior to cDNA second strand synthesis with Klenow Fragment DNA polymerase (New England Biolabs, Beverly, MA, United States) when performing NGS on the Illumina MiSeq instrument (Illumina Inc.) (Colson et al., 2021c), LunaScript RT SuperMix kit (New England Biolabs) when performing NGS with the ONT, or according to the COVIDSeq protocol (Illumina Inc.) following the manufacturer’s recommendations. Generated cDNA was purified using Agencourt AMPure XP beads (Beckman Coulter, Villepinte, France) and quantified using Qubit 2.0 fluorometer (Invitrogen, Carlsbad, CA, United States); fragment sizes were analyzed on an Agilent Fragment analyser 5200 (Agilent Inc., Palo Alto, CA, United States). Next-generation sequencing performed on the NovaSeq 6000 instrument followed the Illumina COVIDSeq protocol (Illumina Inc.), which included first strand cDNA synthesis from extracted viral RNA; cDNA amplification with two COVIDSeq primer pools; fragmentation and tagging of PCR amplicons with adapter sequences; clean up; PCR amplification (seven cycles) of tagmented amplicons; pool, clean up, quantification and normalization of libraries; and library sequencing on a NovaSeq 6000 sequencing system SP flow cell (Illumina Inc.).

### Assembly and Analyses of Genome Sequences

Genome sequences were assembled by mapping on the SARS-CoV-2 genome GenBank accession no. NC_045512.2 (Wuhan-Hu-1 isolate) with the CLC Genomics workbench v.7 (with the following thresholds: 0.8 for coverage and 0.9 for similarity)^11^ as previously described (Colson et al., 2021c), or Minimap2^12^ (Li, 2018). Samtools^13^ was used for soft clipping of Artic primers (see footnote 10) and to remove sequence duplicates (Li et al., 2009). Consensus genomes were generated using the CLC Genomics workbench v.7, and Sam2consensus^14^ through a first in-house script written in Python language^15^. Detection of mutations was performed using the Nextclade tool (see footnote 4), and with freebayes^16^ (Garrison and Marth, 2012) using a mapping quality score of 20 and results filtering by the Python script based on majoritary nucleotide frequencies ≥ 70% and nucleotide depths ≥ 10 [when sequence reads were generated on the NovaSeq Illumina instrument (Illumina Inc.)] or ≥5 [when sequences reads were generated on a MiSeq Illumina instrument (Illumina Inc.)]. SARS-CoV-2 genotyping was performed by a second in-house script written in Python language (see footnote 15) by comparing mutation patterns with those of our database of SARS-CoV-2 variants. Nextstrain clades and Pangolin lineages provided in the present study were determined with the Nextclade web application (see footnote 4) (Hadfield et al., 2018; Aksamentov et al., 2021) and the Pangolin web application (see footnote 5) (Rambaut et al., 2020), respectively. Sequences described in the present study have been deposited on the GISAID sequence database (see footnote 3) (Alm et al., 2020) and can be retrieved online using the GISAID online search tool with “Marseille” as keyword, or selecting sequence names containing “IHU” or “MEPHI.” In addition, they have been deposited on the IHU Marseille Infection website: https://www.mediterranee-infection.com/tout-sur-le-coronavirus/sequencage-genomique-sars-cov-2/.

### Phylogeny Reconstructions and Definition and Naming of SARS-CoV-2 Variants

Phylogeny reconstructions based on the SARS-CoV-2 genomes obtained in our laboratory were performed for genome sequences larger than 24,000 nucleotides obtained with the Illumina technology using the nextstrain/ncov tool^17^ then visualized with Auspice^18^ or FigTree v1.4.4^19^; the limitation of sampling per date of collection of respiratory specimens was deactivated. Within reconstructed trees, we identified and counted all mutations between nodes and between nodes and leaves. This allowed us to define the set of mutations for the most recent ancestor of the clusters corresponding to each of the SARS-CoV-2 variants.

### Numbers of Mutations in SARS-CoV-2 Genomes and Pairwise Genetic Distances for SARS-CoV-2 Genomes Over Time

To compare the numbers of mutations in SARS-CoV-2 genomes and the pairwise genetic distances for these genomes over time, the numbers of nucleotide changes in the SARS-CoV-2 genomes and of amino acid changes in the spike protein relatively to the genome of the Wuhan-Hu-1 isolate (GenBank accession no. NC_045512.2) were obtained using the Nextclade tool (see footnote 4) (Hadfield et al., 2018; Aksamentov et al., 2021) for SARS-CoV-2 genomes used in the phylogenetic analyses. Pairwise nucleotide distances for SARS-CoV-2 genomes were computed using the MEGA7 software v10.2.5^20^. The numbers of nucleotide and amino acid changes and the pairwise nucleotide distances were plotted using the GraphPad software v5.01^21^. The mean numbers of mutations in viral genomes and the mean pairwise genetic distances between genomes over time since SARS-CoV-2 emergence in February 2020 were compared using R 4.0.2 (see footnote 8) and GraphPad software v5.01 (see footnote 21). A *p* < 0.05 was considered statistically significant.

### Real-Time Reverse Transcription-PCR Specific of Marseille Variants

For specimens with Ct values > 30 or those with Ct values < 30 but from which genome sequences were not obtained, we identified those harboring specific Marseille genotypes using qPCR targeting variant-specific regions. We previously described qPCR specific of the Marseille-1 variant (Colson et al., 2021c) and the Marseille-4 variant (Fournier et al., 2021). We used other in-house variant-specific qPCR systems for which the sequences of primers and probes are as follows, provided in 5′-3′ orientation. For the Beta (B.1.351) variant, qPCR targeted the envelope gene and used forward primer C_SA_3_MBF: TGAATTGCAGACACCTTTTGA, reverse primer C_SA_3_MBR: CAACCCTTGGTTGAATAGTCTTG, and probe C_SA_3_MBP: TGACATCTTCAATGGGGAATGT. For the Gamma (P.1) variant, qPCR targeted the nucleocapsid gene and used forward primer C_Bra_2_MBF: GTCAAGCCTCTTCTCGTTCCT, reverse primer C_Bra _2_MBR: AAAGCAAGAGCAGCATCACC, and probe C_Bra_2_MBP: GCAGCTCTAAACGAACTTCTCCTG. For the Delta (B.1.617.2) variant, two qPCR were used that targeted the envelope gene: the first one used forward primer C_IND_1_MBF: AATCTTGATTCTAAGGTTGGTGGT, reverse primer C_IND_1_MBR: TGCTACCGGCCTGATAGATT, and probe C_IND_1_MBP: TTACCGGTATAGATTGTTTAGGAAGTCT; the second one used forward primer P681_MBF: TGACATACCCATTGGTGCAG, reverse primer P681_MBR: GGCAATGATGGATTGACTAGC, and probe P681_MBP: ATTCTCATCGGCGGGCA. Finally, for the Marseille-2 (B.1.177) variant, qPCR targeted the nsp16 gene and used forward primer C2_2_MBF: CATGGTGGACAGCCTTTGTT, reverse primer C2_2_MBR: CATCTATTTGTTCGCGTGGTT, and probe C2_2_MBP: AATGCCTCATCATCTGAAGCAT. PCR conditions were the same than those described previously in Bedotto et al. (2021b) and Fournier et al. (2021). Finally, we used the Applied Biosystems TaqPath COVID-19 kit (Thermo Fisher Scientific, Waltham, United States) then the TaqMan SARS-CoV-2 mutation assay H69-V70 (Thermo Fisher Scientific) for Alpha (B.1.1.7) variant detection, and TaqMan SARS-CoV-2 mutation assays L452R and P681R (Thermo Fisher Scientific) to detect the Delta (B.1.617.2) variant.

## Data Availability Statement

The datasets presented in this study can be found in online repositories. The names of the repository/repositories and accession number(s) can be found in the article/Supplementary Material.

## Ethics Statement

This study received approval of the Ethical Committee of the Mediterranee Infection institute under reference N°2020-016-3. Access to the patients’ biological and registry data issued from the hospital information system was approved by the data protection committee of Assistance Publique-Hopitaux de Marseille (APHM) and was recorded in the European General Data Protection Regulation registry under number RGPD/APHM 2019-73; the latter authorization allowed us to access the patients’ data retrospectively, according to the French law on “Règlement Général sur la Protection des Données (RGPD)”.

## Author Contributions

DR, PC, and P-EF: conceptualization and original draft preparation. DR, PC, P-EF, and AL: methodology. PC, P-EF, HC, JD, AG-G, LH, CA, LB, MBed, EP, CG, MBey, EB, PD, HT-D, PG, J-CL, MM, PB, PP, FF, MD, BL, and AL: data and investigation. DR: supervision. All authors: review and editing of the manuscript, read and agreed to the published version of the manuscript.

## Conflict of Interest

The authors declare that the research was conducted in the absence of any commercial or financial relationships that could be construed as a potential conflict of interest.

## Publisher’s Note

All claims expressed in this article are solely those of the authors and do not necessarily represent those of their affiliated organizations, or those of the publisher, the editors and the reviewers. Any product that may be evaluated in this article, or claim that may be made by its manufacturer, is not guaranteed or endorsed by the publisher.

## Footnotes

1
https://ourworldindata.org/

2
https://www.ecdc.europa.eu/en/geographical-distribution-2019-ncov-cases

3
https://www.gisaid.org/

4
https://clades.nextstrain.org/

5
https://cov-lineages.org/pangolin.html

6
https://www.who.int/en/activities/tracking-SARS-CoV-2-variants/

7
https://www.mediterranee-infection.com/covid-19/

8
https://cran.r-project.org/

9
https://www.mediterranee-infection.com/

10
https://artic.network/

11
https://digitalinsights.qiagen.com/

12
https://github.com/lh3/minimap2

13
https://www.htslib.org/

14
https://github.com/vbsreenu/Sam2Consensus

15
https://www.python.org/

16
https://github.com/freebayes/freebayes

17
https://github.com/nextstrain/ncov

18
https://docs.nextstrain.org/projects/auspice/en/stable/

19
http://tree.bio.ed.ac.uk/software/figtree/

20
https://www.megasoftware.net/

21
https://www.graphpad.com/
