# Expression of Concern: Analysis of SARS-CoV-2 variants from 24,181 patients exemplifies the role of globalization and zoonosis in pandemics

**DOI:** 10.3389/fmicb.2025.1656738

**Published:** 2025-07-07

**Authors:** Frontiers Editorial Office

Following publication, it was brought to our attention that the ethical approval for this study referred to the same ethics approval number 2020-016-03 that had been used across a series of unrelated published studies (1–10). Additionally, an issue around the potential need for approval from a Comité de Protection de Personnes was raised and remains unresolved.

An investigation conducted by the Comité d'éthique at Aix-Marseille Université confirmed that this study is retrospective and did not require approval from a Comité de Protection de Personnes. The institution stated that the quoted ethics approval 2020-016-03 is a generic opinion concerning the study of genetic diversity and evolution of SARS-COV-2 which is valid for any study on this topic.

Frontiers has not received the necessary documentation to confirm these statements.

Dao, T. L., Hoang, V. T., Nguyen, N. N., Delerce, J., Chaudet, H., Levasseur, A., et al. (2021). Clinical outcomes in COVID-19 patients infected with different SARS-CoV-2 variants in Marseille, France. *Clin. Microbiol. Infect*. 27, 1516.e1–1516. doi: 10.1016/j.cmi.2021.05.029

Hoang, V. T., Colson, P., Levasseur, A., Delerce, J., Lagier, J. C., Parola, P., et al. (2021). Clinical outcomes in patients infected with different SARS-CoV-2 variants at one hospital during three phases of the COVID-19 epidemic in Marseille, France. *Infect. Genet. Evol*. 95:105092. doi: 10.1016/j.meegid.2021.105092

Gautret, P., Houhamdi, L., Nguyen, N. N., Hoang, V. T., Giraud-Gatineau, A., Raoult, D., et al. (2021). Does SARS-CoV-2 re-infection depend on virus variant? *Clin. Microbiol. Infect*. 27, 1374–1375. doi: 10.1016/j.cmi.2021.06.029

Hoang, V. T., Assoumani, L., Delerce, J., Houhamdi, L., Bedotto, M., Lagier, J. C., et al. (2022). Introduction of the SARS-CoV-2 Beta variant from Comoros into the Marseille geographical area. *Travel Med. Infect. Dis*. 46:102277. doi: 10.1016/j.tmaid.2022.102277

Colson, P., Fantini, J., Yahi, N., Delerce, J., Levasseur, A., Fournier, P. E., et al. (2022). Limited spread of a rare spike E484K-harboring SARS-CoV-2 in Marseille, France. *Arch Virol*. 167, 583–589. doi: 10.1007/s00705-021-05331-4

Colson, P., Delerce, J., Burel, E., Beye, M., Fournier, P. E., Levasseur, A., et al. (2022). Occurrence of a substitution or deletion of SARS-CoV-2 spike amino acid 677 in various lineages in Marseille, France. *Virus Genes*. 58, 53–58. doi: 10.1007/s11262-021-01877-2

Colson, P., Delerce, J., Pontarotti, P., Devaux, C., La Scola, B., Fantini, J., et al. (2024). Resistance-associated mutations to the anti-SARS-CoV-2 agent nirmatrelvir: selection not induction. *J Med Virol*. 96:e29462. doi: 10.1002/jmv.29462

Colson, P., Chaudet, H., Delerce, J., Pontarotti, P., Levasseur, A., Fantini, J., et al. (2024). Role of SARS-CoV-2 mutations in the evolution of the COVID-19 pandemic. *J. Infect*. 88:106150. doi: 10.1016/j.jinf.2024.106150

La Scola, B., Lavrard, P., Fournier, P. E., Colson, P., Lacoste, A., Raoult, D., et al. (2021). SARS-CoV-2 variant from India to Marseille: the still active role of ports in the introduction of epidemics. *Travel Med. Infect. Dis*. 42:102085. doi: 10.1016/j.tmaid.2021.102085

Colson, P., Levasseur, A., Delerce, J., Pinault, L., Dudouet, P., Devaux, C., et al. (2021). Spreading of a new SARS-CoV-2 N501Y spike variant in a new lineage. *Clin. Microbiol. Infect*. 27, 1352.e1–1352. doi: 10.1016/j.cmi.2021.05.006

