# Supplementary material for: Analysis of SARS-CoV-2 Variants From 24,181 Patients Exemplifies the Role of Globalization and Zoonosis in Pandemics
Source: Front Microbiol. 2022 Feb 7;12:786233. doi: 10.3389/fmicb.2021.786233 (PMC8859183; doi:10.3389/fmicb.2021.786233)
Supplement: Supplementary file 1 [file Data_Sheet_1.docx]

**Supplementary Material for Article**

Analysis of SARS-CoV-2 variants from 24,181 patients exemplifies the role of globalization and zoonosis in pandemics

Philippe Colson^1,2,3 ¥^, Pierre-Edouard Fournier^1,2,3 ¥^, Hervé Chaudet^1,3,4,5^, Jérémy Delerce^1^, Audrey Giraud-Gatineau^1,3,4,5^, Linda Houhamdi^1^, Claudia Andrieu^1^, Ludivine Brechard^1^, Marielle Bedotto^1^, Elsa Prudent^1^, Céline Gazin^1^, Mamadou Beye^1^, Emilie Burel^1^, Pierre Dudouet^1,2,3^, Hervé Tissot-Dupont^1,2,3^, Philippe Gautret^1,3,4^, Jean-Christophe Lagier^1,2,3^, Matthieu Million^1,2,3^, Philippe Brouqui^1,2,3^, Philippe Parola^1,3,4^, Florence Fenollar^1,3,4^, Michel Drancourt^1,2,3^, Bernard La Scola^1,2,3^, Anthony Levasseur^1,2,3^, and Didier Raoult^1,2,3 *^.

^1^ IHU Méditerranée Infection, 19-21 boulevard Jean Moulin, 13005 Marseille, France; philippe.colson@univ-amu.fr (P.C.); pierre-edouard.fournier@univ-amu.fr (P.-E.F.); herve.chaudet@univ-amu.fr (H.C.); jeremy.delerce@univ-amu.fr (J.D.); audrey.giraud-gatineau@gmail.com (A.G.-G); linda@houhamdi@ap-hm.fr (L.H.); claudia.andrieu1@ap-hm.fr (C.A.); ludivine.brechard@ap-hm.fr (L.B.); marielle.bedotto@gmail.com (M.B.); elsa.prudent@ap-hm.fr (E.P.); celine.gazin@ap-hm.fr (C.G.); bemamadou@gmail.com (M.Be); burel.emilie@hotmail.com (E.B.); pierre.dudouet@ap-hm.fr (P.D.); herve.tissot-dupont@ap-hm.fr (H.T.-D.); philippe.gautret@ap-hm.fr (P.G.); jean-christophe.lagier@univ-amu.fr (J.-C.L.); matthieu.million@univ-amu.fr (M.-M.); philippe.brouqui@univ-amu.fr (P.-B.); philippe.parola@univ-amu.fr (P.-P.); florence.fenollar@univ-amu.fr (F.F.); michel.drancourt@univ-amu.fr (M.D.); anthony.levasseur@univ-amu.fr (A.L.)

^2^ Microbes Evolution Phylogeny and Infections (MEPHI), Institut de Recherche pour le Développement (IRD), Aix-Marseille Univ., 27 boulevard Jean Moulin, 13005 Marseille, France

^3^ Assistance Publique-Hôpitaux de Marseille (AP-HM), 264 rue Saint-Pierre, 13005 Marseille, France

^4^ Vecteurs–Infections Tropicales et Méditerranéennes (VITROME), Institut de Recherche pour le Développement (IRD), Aix-Marseille Univ., 27 boulevard Jean Moulin, 13005 Marseille, France

^5^ French Armed Forces Center for Epidemiology and Public Health (CESPA), camp de Sainte Marthe. BP 40026 Marseille Cedex 02, France

^¥^ Contributed equally

***** Correspondence: didier.raoult@gmail.com (D.R.); Tel.: +33 413 732 401

**Supplementary Figures**

**Supplementary Figure 1. Chronological distribution of SARS-CoV-2 diagnoses by qPCR at IHU Méditerranée Infection institute (A) and mean (±standard deviation) numbers of amino acid substitutions in the SARS-CoV-2 spike protein encoded by SARS-CoV-2 genomes recovered from patients sampled per month (B).**

(A) Chronological distribution of SARS-CoV-2 diagnoses is the mean number of diagnoses by qPCR per sliding window of 7 days and step of one day.

(B) Numbers per month of amino acid substitutions in SARS-CoV-2 spike protein were calculated in reference to the genome of the Wuhan-Hu-1 isolate (GenBank Accession no. NC_045512.2).


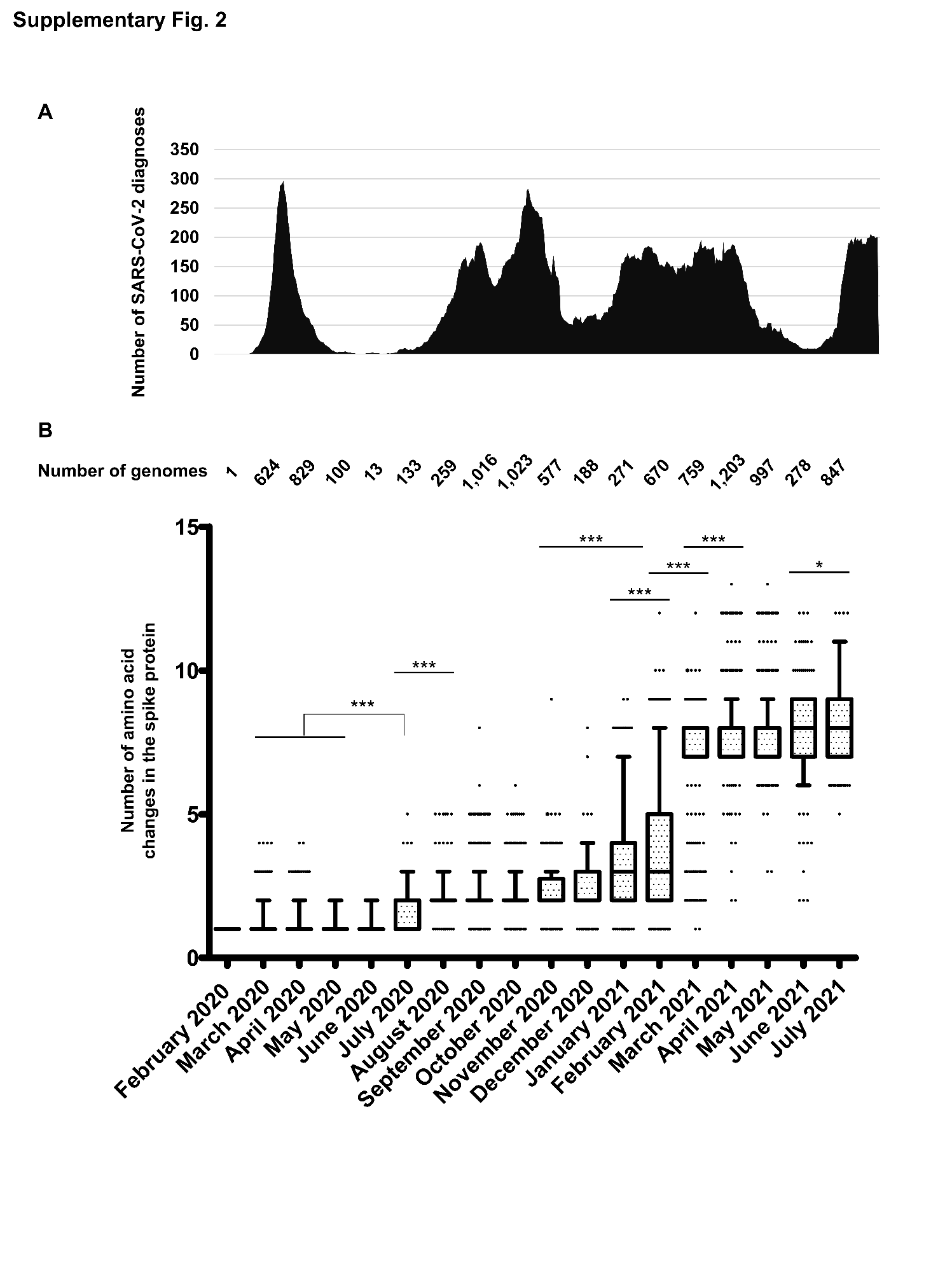


**Supplementary Figure 2. Timeline of trends of searches in Google for world (A) and France (B) and number of publications in Pubmed (C) and BioRxiv or MedRxiv (D) with “variant” and “Covid-19” or “SARS-CoV-2” as keywords as of end of May 2021.**

X-axis indicates time and Y-axis indicate numbers of searches or publications.

Google trends is available at: https://trends.google.fr/trends/?geo=FR

Pubmed is available at: https://pubmed.ncbi.nlm.nih.gov/

BioRxiv is available at: https://www.biorxiv.org/

MedRxiv is available at: https://www.medrxiv.org/

**
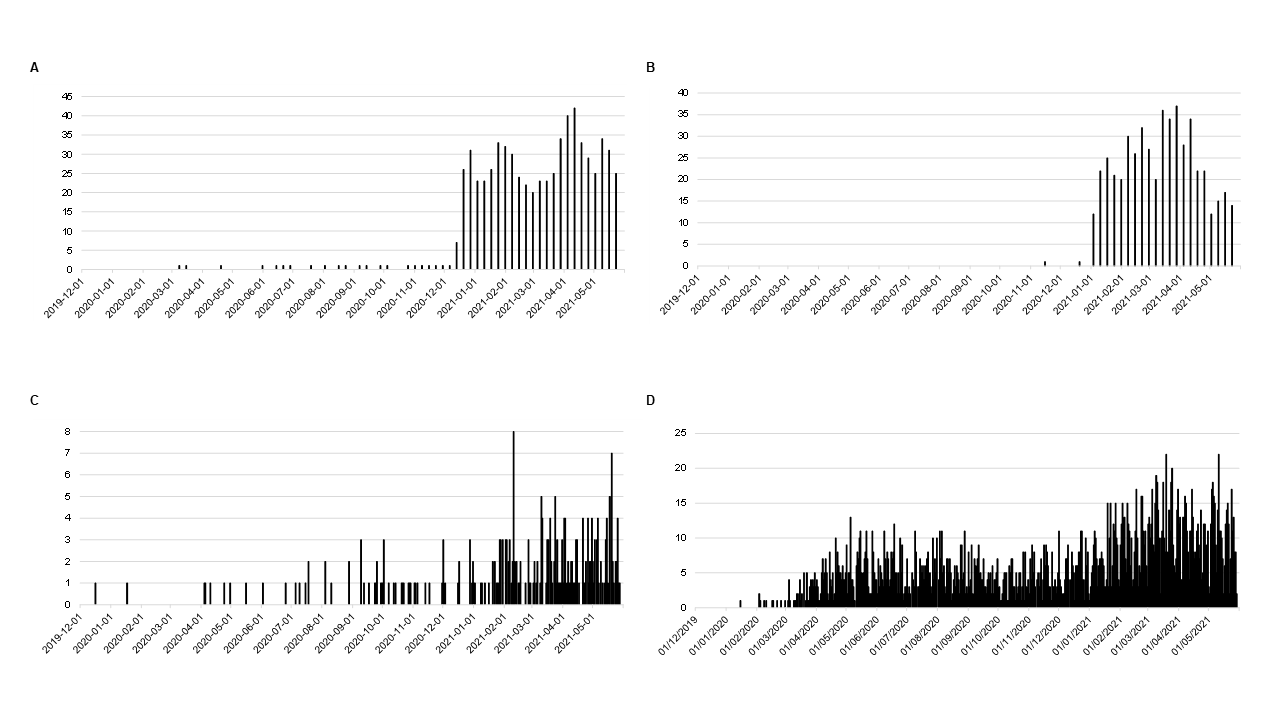
**

**Supplementary Figure 3. Circular phylogenetic tree of 10,773 genomic sequences of SARS-CoV-2 obtained from patients SARS-CoV-2-diagnosed at IHU Méditerranée Infection, Marseille.**

Phylogeny reconstruction based on the SARS-CoV-2 genomes obtained in our laboratory was performed using the nextstrain/ncov tool (https://github.com/nextstrain/ncov) then visualised with FigTree v1.4.4 (http://tree.bio.ed.ac.uk/software/figtree/).


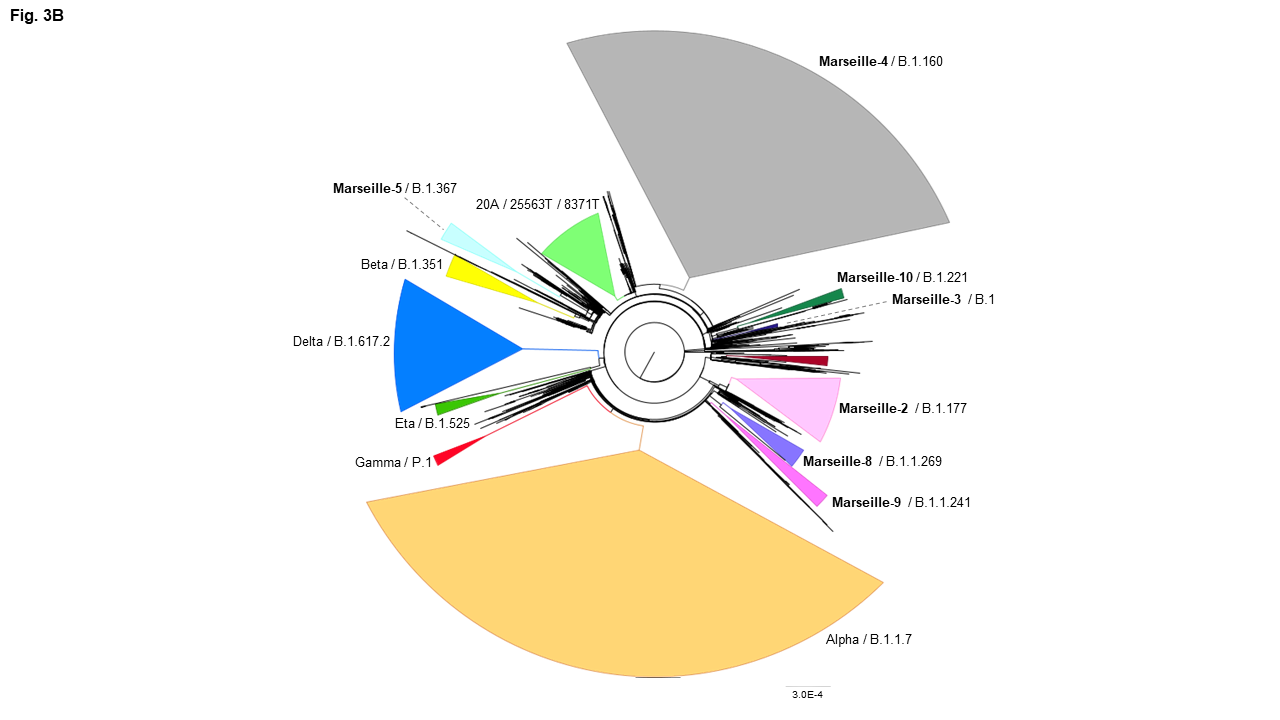


**Supplementary Figure 4. Microarray showing the distribution along the viral genome and in viral genes of nucleotide and amino acid substitutions observed in comparison with the genome of the Wuhan-Hu-1 isolate for the various viral variants detected in 2020 in respiratory samples from patients diagnosed with SARS-CoV-2 infection at IHU Méditerranée Infection.**

Sequences from complete genomes that were obtained were analyzed using the Nextstrain web application (https://clades.nextstrain.org/) (Hadfield et al., 2018; Aksamentov et al., 2021). Representation is adapted from a Nextclade sequence analysis web application output (https://clades.nextstrain.org/).

^a^ In reference to genome GenBank Accession no. NC_045512.2 (Wuhan-Hu-1 isolate); ^b^ Color code for nucleotide mutations: Green: U; yellow: G; blue: C; red: A.

Syn.: synonymous.

See the main manuscript for references Hadfield et al., 2018 and Aksamentov et al., 2021.

**Supplementary Figure 5. Emergence and outcome of the SARS-CoV-2 Marseille-4 variant (according to the GISAID database as of 30 June 2021).**

Number of genomes per day (A), number of genomes per day and country (B), and time range of genome collection and total number of genomes per world region (C) are shown.

GISAID is available at: https://www.gisaid.org/


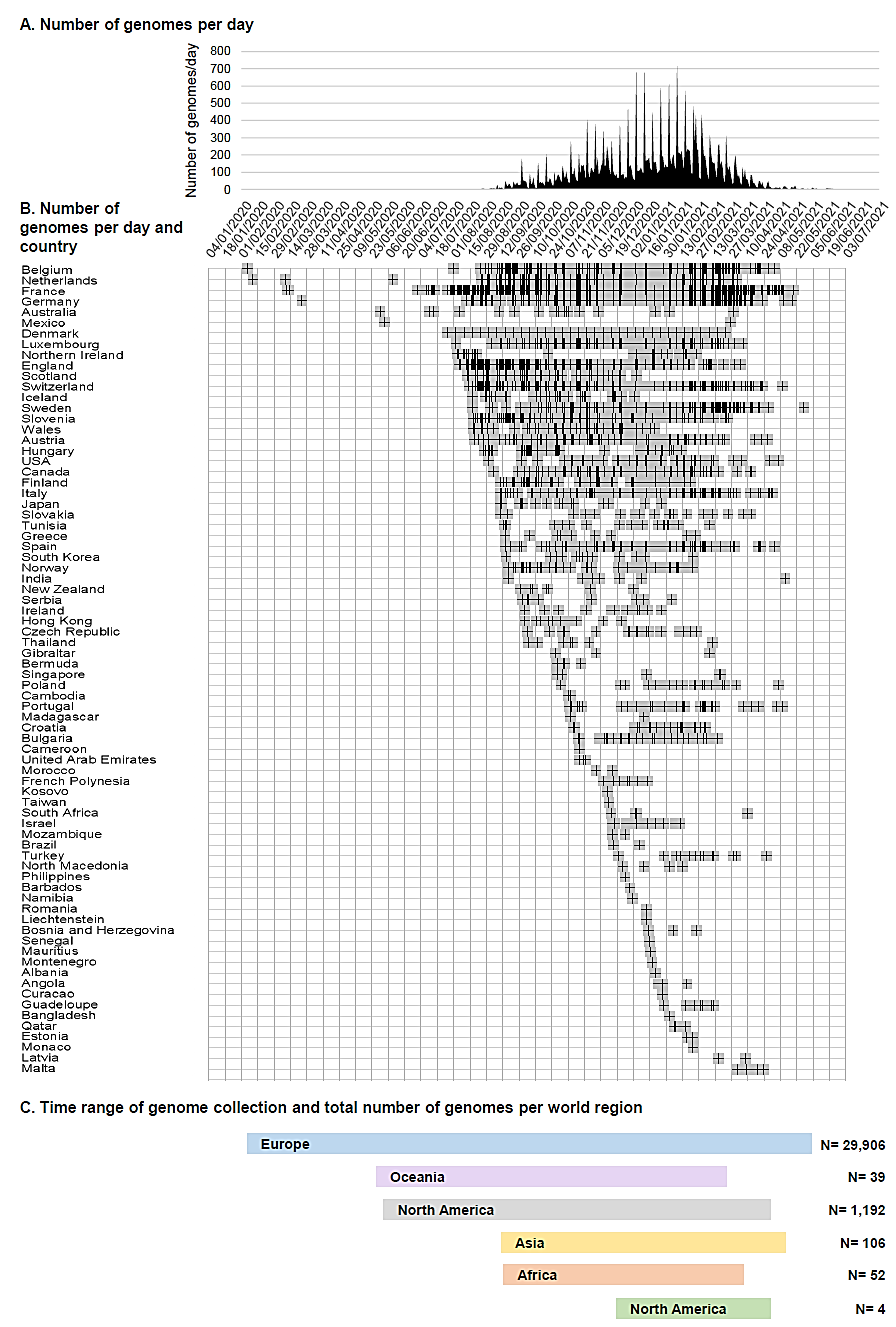


**Supplementary Tables**

**Supplementary Table 1. Median and mean (±standard deviation) numbers per month of nucleotide changes in SARS-CoV-2 genomes and of amino acid substitutions in the spike protein for genomes obtained from patients SARS-CoV-2-diagnosed at IHU Méditerranée Infection, Marseille.**

Numbers of mutations in SARS-CoV-2 genomes and of amino acid substitutions in the spike protein were calculated in reference to the genome of the Wuhan-Hu-1 isolate (GenBank Accession no. NC_045512.2).

|  | February 2020 | March 2020 | April 2020 | May 2020 | June 2020 | July 2020 | August 2020 | September 2020 | October 2020 | November 2020 | December 2020 | January 2021 | February 2021 | March 2021 | April 2021 | May 2021 | June 2021 | July 2021 |
| --- | --- | --- | --- | --- | --- | --- | --- | --- | --- | --- | --- | --- | --- | --- | --- | --- | --- | --- |
| **Number of genomes analyzed** | 1 | 624 | 829 | 100 | 13 | 133 | 259 | 1,016 | 1,023 | 577 | 188 | 271 | 670 | 759 | 1,203 | 997 | 278 | 847 |
|  |  |  |  |  |  |  |  |  |  |  |  |  |  |  |  |  |  |  |
| **Nucleotide changes in the genome** |  |  |  |  |  |  |  |  |  |  |  |  |  |  |  |  |  |  |
| Median | 5 | 8 | 8 | 9 | 11 | 13 | 21 | 21 | 22 | 24 | 26 | 24 | 27 | 34 | 36 | 36 | 37 | 36 |
| Mean | 5.0 | 8.1 | 8.5 | 9.4 | 10.8 | 15.0 | 20.0 | 20.5 | 21.9 | 23.0 | 25.2 | 24.2 | 26.4 | 34.1 | 35.7 | 36.2 | 36.8 | 36.1 |
| Standard deviation | 0.0 | 2.0 | 2.0 | 2.3 | 2.7 | 4.4 | 5.0 | 4.7 | 4.1 | 4.1 | 4.3 | 5.2 | 5.7 | 4.2 | 3.8 | 3.9 | 4.7 | 3.1 |
|  |  |  |  |  |  |  |  |  |  |  |  |  |  |  |  |  |  |  |
| **Amino acid substitutions in the spike** | |  |  |  |  |  |  |  |  |  |  |  |  |  |  |  |  |  |
| Median | 1 | 1 | 1 | 1 | 1 | 2 | 2 | 2 | 2 | 2 | 2 | 3 | 3 | 7 | 7 | 8 | 8 | 8 |
| Mean | 1.0 | 1.1 | 1.1 | 1.1 | 1.2 | 1.7 | 2.2 | 2.2 | 2.2 | 2.3 | 2.5 | 3.3 | 3.8 | 7.1 | 7.7 | 7.8 | 7.8 | 8.2 |
| Standard deviation | 0.0 | 0.5 | 0.4 | 0.3 | 0.4 | 0.8 | 0.7 | 0.7 | 0.7 | 0.7 | 1.0 | 2.0 | 2.2 | 1.3 | 1.0 | 1.1 | 1.5 | 1.5 |

**Supplementary Table 2. Monthly numbers of genome sequences, spike sequences and qPCR performed each month, and proportions of positive diagnoses by qPCR of SARS-CoV-2 infection as of July 2021.**

| **Month/Year** | **Total number of positive diagnoses** |  | **SARS-CoV-2 genotyping approach** | | | | | | | |
| --- | --- | --- | --- | --- | --- | --- | --- | --- | --- | --- |
|  |  |  | **Next-generation genome sequencing** | |  | **Next-generation spike gene fragment sequencing** | |  | **qPCR** | |
|  |  |  | **Number of genomes** | ***% of the positive diagnoses*** |  | **Number of sequences** | ***% of the positive diagnoses*** |  | **Number of positive** | ***% of the positive diagnoses*** |
| 2020/2 | 6 |  | 3 | *50* |  | 1 | *17* |  | 0 | *0,0* |
| 2020/3 | 3,731 |  | 777 | *21* |  | 1 | *<0,1* |  | 7 | *0,2* |
| 2020/4 | 2,790 |  | 877 | *31* |  | 2 | *0,1* |  | 5 | *0,2* |
| 2020/5 | 207 |  | 70 | *34* |  | 0 | *0,0* |  | 1 | *0,5* |
| 2020/6 | 48 |  | 23 | *48* |  | 0 | *0,0* |  | 0 | *0,0* |
| 2020/7 | 368 |  | 244 | *66* |  | 0 | *0,0* |  | 2 | *0,5* |
| 2020/8 | 2,620 |  | 741 | *28* |  | 1 | *<0,1* |  | 15 | *0,6* |
| 2020/9 | 4,664 |  | 1,192 | *25* |  | 1 | *<0,1* |  | 16 | *0,3* |
| 2020/10 | 6,413 |  | 1,713 | *27* |  | 0 | *0,0* |  | 19 | *0,3* |
| 2020/11 | 3,911 |  | 751 | *19* |  | 1 | *<0,1* |  | 26 | *0,7* |
| 2020/12 | 2,116 |  | 450 | *21* |  | 1 | *0,1* |  | 213 | *10* |
| 2021/1 | 4,391 |  | 692 | *16* |  | 50 | *1* |  | 2,445 | *56* |
| 2021/2 | 5,928 |  | 1,075 | *18* |  | 992 | *17* |  | 2,751 | *46* |
| 2021/3 | 6,016 |  | 724 | *12* |  | 2,639 | *39* |  | 1,879 | *31* |
| 2021/4 | 6,210 |  | 1,503 | *24* |  | 0 | *0,0* |  | 3,667 | *59* |
| 2021/5 | 2,566 |  | 1,137 | *44* |  | 0 | *0,0* |  | 1,199 | *47* |
| 2021/6 | 660 |  | 278 | *42* |  | 0 | *0,0* |  | 293 | *44* |
| 2021/7 | 3,767 |  | 1,412 | *37* |  | 0 | *0,0* |  | 1,674 | *44* |

**Supplementary Table 3. Weekly proportions of the variants among patients SARS-CoV-2-diagnosed in our institute.**
